# Comparing the effects of a commercial and a prototype vitrification medium on meiotic spindle morphology and survival rate of mouse oocytes

**DOI:** 10.5935/1518-0557.20210117

**Published:** 2022

**Authors:** Iara Gonçalves Roberto Viana, Alessandra Aparecida Vireque, Paula Andrea Navarro

**Affiliations:** 1 Division of Human Reproduction, Department of Gynecology and Obstetrics, Ribeirao Preto Medical School, University of São Paulo, Ribeirao Preto, SP, Brazil; 2 Clinic Semear Fertility, Ribeirao Preto, SP, Brazil; 3 Invitra Assisted Reproductive Technologies LTDA., Ribeirao Preto, SP, Brazil; 4 National Institute of Hormones and Women’s Health CNPq, Brazil

**Keywords:** oocyte, vitrification medium, survival, meiotic normality

## Abstract

**Objective:**

To compare oocyte survival and meiotic spindle normality between vitrified-warmed oocytes in a mouse embryo assay using Tvitri-4 or Ingámed vitrification media.

**Methods:**

C57BL/6 female mice aged 8-12 weeks were submitted to superovulation with pregnant mare’s serum gonadotropin and human chorionic gonadotropin (hCG) for obtaining of *in vivo* matured oocytes. The oocytes were randomly distributed into one of the following three groups: CTR - control (fresh oocytes); ING - oocytes vitrified-warmed in a standard commercial kit supplied by Ingámed, and T4 - oocytes vitrified-warmed in the novel prototype Tvitri-4 medium. After warming and recovery culture, oocytes were assessed with respect to survival rate (SR) and both meiotic spindle morphology and chromosome alignment of each oocyte fixed in the sagittal position after immunostaining and analysis by confocal microscopy.

**Results:**

A total of 354 mature oocytes were vitrified in ING (n=178) and T4 (n=176), out of which 299 (85%) survived after warming. Oocyte survival rates were not statistically different (p=0.08) between ING (145/178=81.5%) and T4 (154/176=87.5%). Regarding meiotic normality, there were no significant changes in the proportion of oocytes with normal meiotic spindle morphology and chromosome structure between ING (52,2%) and T4 (63.4%) after warming (RR: 0.95, 95% CI: 0.92-1.607). When the meiotic normality was assessed using the CTR group as a reference in the analysis of relative risk, no significant differences were observed between T4 (63.4%) and CTR (70.5%) (RR: 0.95, 95% CI: 0.72-1.12). On the other hand, the percentage of oocytes retaining normal meiotic spindle morphology and chromosome configuration in ING (52.2%) was lower than in the CTR group (RR: 0.95, 95% CI: 0.57-0.97).

**Conclusions:**

The novel prototype Tvitri-4 medium was efficient in preserve survival rate and meiotic spindle normality of oocytes and, with further verification, may be able to replace commercially available media in future clinical applications.

## INTRODUCTION

Oocyte vitrification had its clinical use in the United States of America recommended in 2013 leading to the introduction of oocyte vitrification technique (VF) in an increasing number of *in vitro* fertilization (IVF) clinics worldwide (Argyle *et al*., 2016). It was only with the beginning of VF use that oocyte post-warming parameters reached standards compatible with its clinical introduction. This was highlighted by a 2014 Cochrane review, which reported that VF increased oocyte survival by 29%, and fertilization by 19% when compared to slow freezing (SF) (Glujovsky *et al*., 2014). A study evaluating 3610 vitrified oocytes, reported a 90% survival rate (SR), translating to a clinical pregnancy rate of 48%, and the overall vitrified-warmed oocyte to live born child efficiency of only 6.5% (Cobo *et al*., 2015). This data indicates that damages to the female gamete cannot be completely avoided, even VF resulting in less severe cell stress (Tatone *et al*., 2010) compared to the SF technique (Cil *et al*., 2013; Oktay *et al*., 2006).

Highly concentrated cryoprotectant solutions added to VF media allow a sudden reduction in temperature, allowing the transition from a liquid to a glassy state, thus preventing the ice crystals formation (Sansinena *et al*., 2011). However, repairing the damages induced by cryopreservation in cell structure and function requires the generation of energy that results in an increased production of reactive oxygen species (ROS) (Tatone *et al*., 2010). Thus, the absence of crystallization does not exclude the possibility of cell damages resulting from oxidative stress (Gupta *et al*., 2010; Somfai *et al*., 2007) and, although re-crystallization episodes are not frequently observed during the VF step, it can occur during the warming process (Luyet, 1937).

The highly organized structure of the oocyte and its collection of transcripts, proteins, and energetic substrates, that support cleavage until embryonic genome activation occurs, make this cell prone to various unphysiological alterations after vitrification (Elnahas *et al*., 2010; Sirard, 2012; Tamura *et al*., 2013). For instance, any damage to oocyte chromatin structure may result in substantial deleterious defects in the developmental competence of the embryo. Indeed, the meiotic spindle plays a critical role on the generation of a fertilized oocyte that contains the correct number and set of chromosomes (Dumont & Desai, 2012; Gadde & Heald, 2004). In addition, further evidence has highlighted that a recovery ability of the spindle reassembly is a requirement for correct chromosome alignment and segregation after fertilization (Gomes *et al*., 2008). Therefore, we hypothesize that different VF media and different protocols can interfere with the spindle conformation. Although the oocyte VF represents one of the great advances in assisted reproductive technologies (ART) for providing live births rates similar to those using fresh oocytes in some groups of patients, it is important to consider that VF is a recent technique and most studies related to the safety of the method involve perinatal, neonatal, and short-term data, with a lack of data about the safety of the method in the medium and long term (Cobo *et al*., 2014).

A successful vitrification media consisting of 15% EG, 15% DMSO and 0.5 mol/l sucrose and two media used for warming with a sucrose concentration of 1.0 and 0.5 mol/l, respectively, was described by Kuwayama *et al*. (2005). Since then, advances in vitrification technology were more focused on vitrification protocol adjustments and novel cryo devices rather than the vitrification media. Considering specific characteristics of the oocytes such as the size and its relationship with the dehydration and rehydration processes, and also the membrane permeability (Smith *et al*., 2011), improvements in vitrification media should take into account the importance of protecting the membrane lipid bilayer. In this way, it seems that trehalose is more efficient in stabilize the membrane lipid bilayer and/or increase the glass transition temperature compared to sucrose used as non-permeant cryoprotectant. It has been previously suggested that trehalose seems to have an important role by preventing membrane fusion and aggregation of intramembranous particles and also by stabilizing bilayers during reduced water states (Ishida *et al*., 1997; Rudolph & Crowe, 1985).

The number of potential combinations of cryoprotective agents in various proportions into vitrification and warming solutions is nearly infinite. Besides, the development of better vitrification solutions requires systematic knowledge concerning the mechanisms and modulators of the toxicity of cryoprotectant agents. Designing of improved solutions for oocyte cryopreservation, therefore, will demand prototyping and research. Prototyping culture media are offered worldwide by facilities in the IVF market and are an important alternative for advances in research and improvement of ART procedures. Tvitri-4 is a prototype produced on small scale for research by the startup Invitra®, based on standard cryopreservative compositions with four modifications including a high-purity low endotoxin (HPLE) trehalose instead sucrose, reduced non-permeant cryoprotectant concentration, and addition of two amino acids (AA) as supplemental penetrating cryoprotectants and membrane protein stabilizers.

In this context, studies using prototype vitrification media for research, and experimental models are important to investigate the safety of the vitrification-warming processes and the role of different cryoprotectant formulations on minimize damage to membranes, cytoskeleton, and genetic content induced by the VF process. Thus, the objective of this study was to compare oocyte survival and meiotic spindle normality between mouse vitrified-warmed oocytes using two distinct formulations developed in Brazil: Tvitri-4 or Ingámed, a standard vitrification/warming media already validated and commercially available in Brazil.

## MATERIAL AND METHODS

For this study, 6-12-week-old C57BL/6J wild-type female and 8-months-old male mice were housed in the mouse vivarium of the University of São Paulo/Ribeirao Preto Medical School according to the animal care guidelines. The study was approved by the Ethics Committee on Animal Use (CEUA) of University of São Paulo (Opinion No. 004.07/CEUA-USP).

### Experimental design

In order to compare SR and the percentage of meiotic normality of mouse oocytes vitrified in Tvitri-4 or Ingámed medium, mouse oocytes were randomly distributed in three experimental groups:

CTR: fresh control

ING: vitrification/warming in standard commercial kit supplied by Ingámed (Ingamed Ltda, Perobal, PR, Brazil)

T4: vitrification/warming in the prototype Tvitri-4 medium

#### Preparation of Tvitri-4 media

Unless stated otherwise, all of the chemicals used were purchased from Sigma-Aldrich (St. Louis, MO, USA). The base medium for the preparation of oocyte vitrification solutions was buffered with 4- (2hydroxyethyl) piperazine-1-ethanesulphonic acid (HEPES), NaHCO_3_ and human albumin supplement. The equilibration solution (ES) was prepared with base medium containing the permeant cryoprotectants dimethyl sulfoxide (7.5% v/v), ethylene glycol (7.5% v/v), gentamicin, protein supplement, and two amino specific acids (AA). The vitrification solution was prepared with the base medium containing dimethyl sulfoxide (15% v/v) and ethylene glycol (15% v/v), gentamicin, protein supplement, AA, and 0.3M HPLE trehalose as non-permeant cryoprotectant. The warming solution was prepared with the base medium supplemented with 0.3M trehalose, and AA. The dilution solution (DS) and washing solution (WS) were prepared with the base medium supplemented with 0.15M or 0.0M trehalose, respectively, and AA. The base medium was homogenized by centrifugation at 10,000 x *g*, the pH and osmolarity adjusted, and membrane filtered. All solutions were aseptically processed in an ISO Class 5 laminar flow cabinet.

#### Formulation of Ingámed media

According to the manufacturer, the composition of equilibration and vitrification solutions (VI-1 and VI-2, respectively) comprises buffered culture medium, protein supplement, ethylene glycol, dimethyl sulfoxide, sucrose, and gentamicin. Warming solutions are composed by DV-I, DV-2, and DV-III bottles containing buffered culture medium, protein supplement and sucrose. Probably, DV-III corresponds to washing solutions (WS) and is therefore devoid of sucrose.

#### Superovulation protocol

Female mice were superovulated by intraperitoneal injection of 5 IU equine chorionic gonadotropin (eCG; Novormon - Syntex SA - Buenos Aires, Argentina), followed by 5 IU human chorionic gonadotropin (hCG; Chrorulon - Syntex SA, Buenos Aires, Argentina) 48 h later.

#### Oocyte Collection

Approximately 14 hours after hCG administration, the females were euthanized by cervical dislocation and oocytes were collected from oviducts using Embryomax M2 medium (EMD Millipore Corporation, Billerica, MA USA). The oocyte-cumulus complexes (COCs) were subjected to the denudation process, approximately 1 hour after collection, using hyaluronidase (Ingase 80 IU / ml, Ingamed, Maringá-RS) in HTF medium. After denudation, all oocytes that had good morphology, namely no indication of degeneration, were randomly divided between groups according to the experiment. Oocytes from ING and T4 groups were then transferred to dishes containing KSOM + AA medium (EMD Millipore Corporation, Billerica, MA USA) pre-equilibrated and covered with mineral oil, until the time of vitrification.

#### Vitrification and warming of oocytes

Oocytes were vitrified in T4 media following a standard protocol (Vitrification Kit media, Irvine, USA) (Irvine Scientific, 2019), and using aseptic devices (Cryo-Inga, Ingamed Ltda, Perobal, PR, Brazil). Four oocytes were vitrified in each straw. For vitrification, oocytes were placed in 20 µL drop of HTF medium for 1 min. After that, oocytes were gradually exposed to the 20 µL drops of ES as follows: two minutes in ES-1, two minutes in ES-2, and three minutes in ES-3. Afterwards, they were transferred from ES-3 to three consecutive 20 µL drops of vitrification solution (VS) for 50 s before loading. Then, oocytes were loaded in Cryo-Inga devices and immersed into the liquid nitrogen (LN2). The total time from when the oocyte was placed into the vitrification solution until its immersion into LN2 was between 60 and 90 s. For warming, Cryo-Inga straws were taken out of the liquid nitrogen, the tips with oocytes were quickly placed in a 250 µL drop of prewarmed thawing solution (TS) at 37°C for 1 minute, and gently agitated for 3 s. Then, oocytes were transferred to the 50 µL drop of dilution solution (DS) for 4 minutes and finally to two 25 µL drops of washing solution (WS-1 and WS-2) for 4 min each at room temperature.

**Figure 1:**
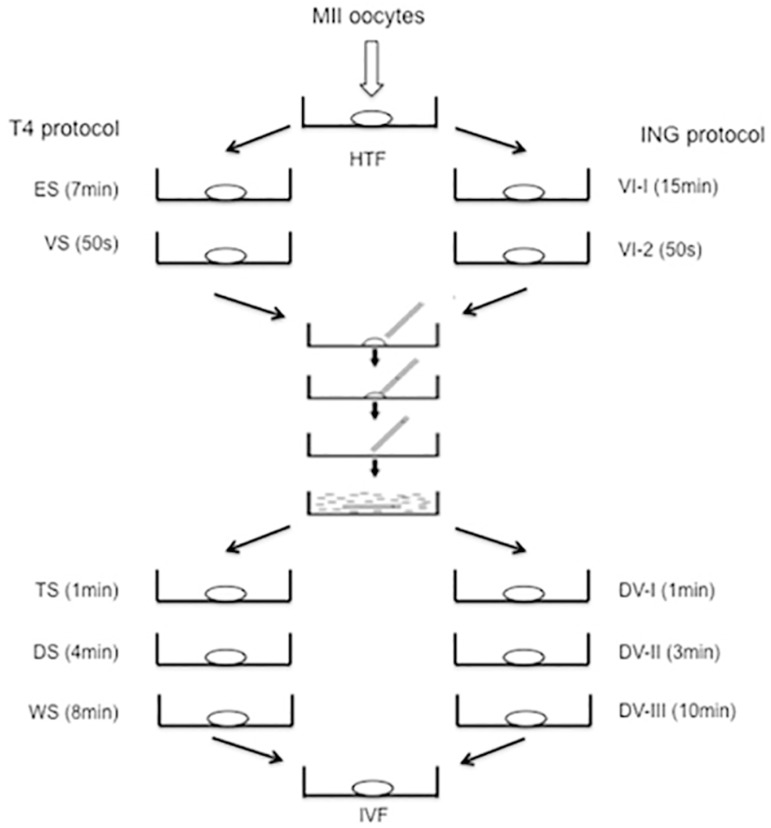
Protocols of vitrification and warming of oocytes to T4 and ING groups. **Note:** Oocyte vitrification procedure. Mouse oocytes were transferred to HTF medium regardless of the experimental group. Vitrification phase: T4 protocol (left) comprised the oocyte exposure to equilibration solution 1 (ES-20 µL drops) and vitrification solution 2 (VS - 20 µL drop); the ING protocol (on the right side) included the vitrification solution 1 (VI-1 - 20 µL drop) and vitrification solution 2 (VI-2- 20 µL drop). After loading using the open pulled straw (OPS) protocol, oocytes were immersed in liquid nitrogen until 90s. Warming phase: oocytes were warmed by T4 protocol (left) using a thawing solution 1 (TS - 250 µL drop), dilution solution (DS - 50 µL drop) and washing solution (WS - 25 µL drop); ING protocol (on the right side) comprised a thawing solution 1 (DV-I - 250 µL drop), thawing solution 2 (DV-II - 100 µL drop) and DV-III (100 µL drop). After warming, the next step was the recovery culture (incubation in IVF culture medium with 20 % (v/v) protein supplement for 2 h at 37 °C). HTF = Human Tubal Fluid medium; T4 = Tvitri-4 medium; ING = Ingámed medium.

Regarding ING oocytes, vitrification and warming were carried out as previously described (Almodin *et al*., 2010; Nishikawa *et al*., 2010) and four oocytes were vitrified en each straw. For vitrification, oocytes were placed in 20 µL drop of HTF medium and were gradually exposed to the 20 µL drops of VI-1 solution for 3 min as follows: three minutes in VI-1, two minutes in ES-2, and three minutes in ES-3. Afterwards, they were transferred to 20 µL drop of VI-1 solution, in which they were equilibrated for 9 min. After this period, the four oocytes were immersed into three consecutive 20 µL drops of VI-2 vitrification solution and placed into the hole in the tip of a Cryo-Inga strip with a minimum amount of vitrification solution, and then immediately immersed into LN2. When more than 0.1 µL of solution was placed with the oocyte on the strip, excess liquid was aspirated. The total time from when the oocyte was placed into the vitrification solution until its immersion into LN2 was between 50 and 60 s. Immediately after removing the Cryo-Inga strip from the protecting plastic sheath, the thin part of the strip was totally immersed into 250 µL drop of DV-1 warming solution at 37ºC for 1 min. The oocyte was then transferred to 100 µL drop of DV-II diluting sucrose solution for 3 min at room temperature and rinsed in a 100 µL drop of buffer solution DV-III twice, for 5 min each. The warmed oocytes from ING or T4 groups were then transferred to a previously equilibrated IVF culture medium with 20 % (v/v) protein supplement for 2 h. These procedures are illustrated in Figure 1.

#### Oocyte survival after warming

Oocytes that showed clear bright homogeneous cytoplasm and an intact plasma membrane and zona pellucida, and normal-sized perivitelline space were considered to have survived the vitrification (Almodin *et al*., 2010). The SR after warming represents the number of survived oocytes divided by the total number of vitrified oocytes multiplied by 100 and were calculated only for oocytes vitrified at the MII stage.

#### Analysis of meiotic spindle morphology and chromosome configuration

**Figure 2:**
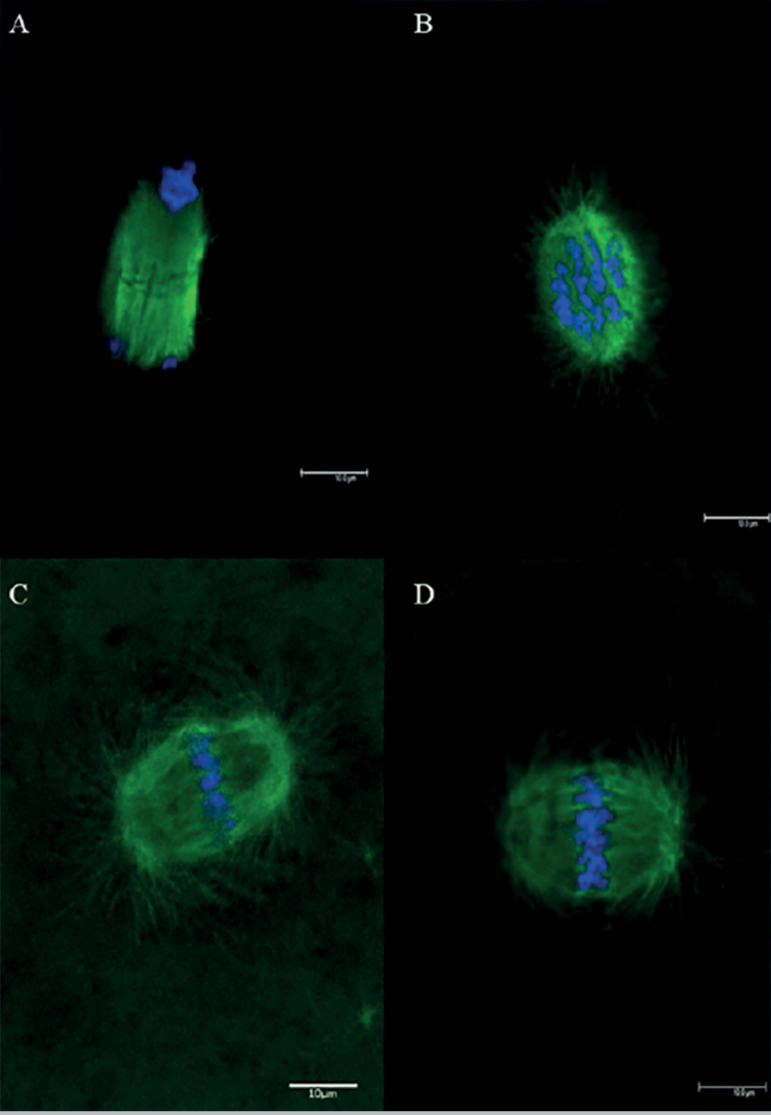
Nuclear maturation stages of murine oocytes matured in vivo and visualized by confocal microscopy. **Note:** (A) telophase I (TI) stage; (B) meiotic spindle viewed in a polar position (not analyzable); (C and D) meiotic spindle in a sagittal position (analyzable). Green: spindle marked with anti-β-tubulin antibody and secondary antibody conjugated with fluorescein isothiocyanate; blue: chromosomes marked with DAPI. Scale bar = 10 µm. **Note:** Comparisons between groups was performed with chi-square test. Fixed = total number of oocytes per group; MI or TI = immature oocytes, in metaphase or telophase I stage among the 3 groups analyzed (*p*=0.2802); MII = mature oocytes, in metaphase II. Analyzable MII: MII oocytes whose cell spindle was fixed in a lateral or sagittal position (microfilaments longitudinal position, making it possible to visualize the two poles of the meiotic spindle) and the chromosomes arranged in line in the median region of the spindle among the 3 groups (*p*=0.9506).

Oocytes were fixed and extracted for 30 min at 38.5°C in a microtubule-stabilizing buffer, as previously described (Ferreira *et al*., 2009; Liu *et al*., 1998). The oocytes were washed extensively, blocked overnight at 48°C in wash medium (PBS supplemented with 0.02% NaN3, 0.01% Triton X-100, 0.2% non-fat dry milk, 2% goat serum, 2% BSA and 0.1 M glycine) and incubated overnight at 48^°^C with mouse monoclonal anti-b-tubulin antibody (1:1000). Oocytes were washed and incubated with fluorescein isothiocyanate-conjugated anti-mouse IgG (1:500; Zymed Laboratories, Invitrogen Corporation, Carlsbad, CA, USA) for 2 hours at 38.5°C, washed again and then incubated with rhodamine-phalloidin (1:1000; Molecular Probes, Invitrogen Corporation, Eugene, OR, USA). After further washing, the samples were stained for DNA with Hoechst 33342 (10 mg/ml) in Vectashield mounting medium (H-1000, Vector, Burlingame, CA, USA) on a glass slide and sealed. The samples were assessed under a high-performance confocal microscope (Confocal Leica TCS SP5, Leica Microsystems, Mannheim, Germany). Oocytes were classified as being in metaphase I, anaphase I, telophase I (Figure 2A) and metaphase II (Figures 2B,2C, 2D). For better accuracy, oocytes at MII stage were subdivided into analyzable and non-analyzable. MII oocytes were considered analyzable when the meiotic spindle could be visualized in a lateral or sagittal position (microfilaments longitudinal position, being possible to visualize the two poles of the meiotic spindle) and the chromosomes arranged in line in the median region of the spindle (Figure 2C and 2D). They were considered non-analyzable when the spindle was observed in a polar position (Figure 2B) (Ju *et al*., 2005), which prevents a detailed view of the spindle. Analyzable MII oocytes were classified as “normal” when they presented a barrel-shaped meiotic spindle with microtubules organized from one pole to another, chromosomes aligned on the metaphasic plate at the spindle equator and the presence of a CP; or considered “abnormal” when they presented an altered meiotic spindle (reduced longitudinal dimension, disorganized or missing microtubules) and/or altered chromosomal configuration (dispersed or displaced from the metaphasic plate).

### Statistical analysis

An exploratory analysis of the data was performed using absolute and relative frequencies. The comparison between groups and outcomes (survival and meiotic normality) was performed considering the chi-square test. In situations where the frequency was less than five, Fisher’s exact test was considered. The tests were performed using the SAS version 9.4 program. The relative risk followed by the confidence interval was estimated to quantify the possible association between the groups. It was considered statistically relevant when the confidence interval does not contain a value of 1. We set the significance level at 5%.

## RESULTS

A total of 354 oocytes were vitrified in ING (n=178) and T4 groups (n=176), with a total of 299 (85%) oocytes surviving after warming. Oocyte survival rates were not statistically different (*p*=0.08) between ING (145/178=81.5%) and T4 (154/176=87.5%) (Table 1).

**Table 1:** Comparison of survival and meiotic normality between the experimental groups.

Group	Oocytes thawed	Oocytes survived	Survival rates% (RR; IC 95%)	MII oocytes with analyzable spindle	Normal MII oocytes	Meiotic normality rate % (RR; IC 95%)
Fresh	N/A*	N/A	N/A	78	55	70.5 (REF)^†^
ING	178	145	81.5 (REF)	69	36	52.2 (0.740; 0.566-0.967)
T4	176	154	87.5 (1.09; 0.997-1.192)	82	52	63.4 (0.899; 0.723-1.119)
Total	354	299	84.5	229	143	62.4

Regarding meiotic spindle and chromosomal configuration, a total of 424 oocytes were fixed, divided into CT (n=149), ING (n=133) and T4 (142) groups. There were no differences in the percentages of immature (MI and TI) and mature oocytes among the 3 groups analyzed (*p*=0,2802). In addition, no differences were observed in the rates of analyzable MII oocytes among the 3 groups (*p*=0.9506) (Table 2). Technical issues, such as oocyte degeneration, oocyte loss during passage from one well to another and oocyte loss during slide assembly, during the fixation protocol caused the loss of some eggs, totaling for ING n=12 and T4 n=13.

**Table 2:** Oocytes total / group fixed, classified: MI or TI and MII, and Analyzable.

Group	Oocytes			
	Fixed	MI ou TI (%)	MII (%)	Analyzable MII (%)
**Fresh**	**149**	**47 (31.5)**	**102 (68.5)**	**78 (76.5)**
**ING**	**133**	**45 (33.8)**	**88 (66.2)**	**69 (78.4)**
**T4**	**142**	**36 (25.4)**	**106 (74.6)**	**82 (77.4)**
Total	424	128 (30.2)	296 (69.8)	229 (77.4)

**Note:** Comparisons between groups was performed with chi-square test. *p* value <0.05. The relative risk (RR) followed by the confidence interval was estimated to quantify the possible association between the groups. Data presented as percentage; relative risk and confidence interval (%; RR; IC 95%).

* N/A: not analyzed.

† REF is the control.

When the meiotic normality rates were compared using CTR group as a reference in the analysis of relative risk, no significant differences between T4 (63.4%) and CTR (70.5%) (RR: 0.95, 95% CI: 0.72-1.12) were observed. However, the percentage of meiotic normality was significant lower in ING (52.2%) compared to CTR (RR: 0.95, 95% CI: 0.57-0.97) (Table 1). When the two vitrification media were compared to each other, using ING group as a reference in the analysis of relative risk, not significant differences were detected in meiotic normality rates (RR: 0.95, 95% CI: 0.92-1.607) (Table 1).

## DISCUSSION

In this preliminary validation study, two different vitrification and warming media were compared regarding SR and meiotic spindle normality in oocyte cryopreservation using a mouse embryo assay. Vitrification has been successfully used for cryopreservation of human oocytes and there are various cryosolutions which contain distinct formulation including basal medium, human serum albumin supplement, and different combinations and concentrations of permeant and non-permeant cryoprotectants. In the present study, differences in the composition of the two vitrification media assessed had no impact on oocyte survival rates. On the other hand, a slight difference in the meiotic spindle normality was detected when T4 or ING were compared to CTR, with T4 retaining rates of proper alignment of chromosomes and spindle integrity similar to fresh oocytes. Since mouse oocytes have been used as translational model for evaluating new IVF medical devices before their clinical use in human oocytes (Baumann *et al*., 2007), our findings demonstrated the potential of Tvitri-4 media for human oocyte vitrification.

Although oocyte vitrification is an established technique in the IVF laboratories with comparable results to fresh cycles in some groups of patients there is still room from large scale trials to clarify its possibly “cloaked” negative impact on oocyte physiology (Potdar *et al*., 2014; Rienzi *et al*., 2017). Among reproductive cells and tissues, metaphase II oocytes are notably vulnerable to cryopreservation, mainly because of their large size, low surface area to volume ratio, relatively high-water content, membrane permeability and a set of labile structures comprising the meiotic spindle (Iussig *et al*., 2019; Smith *et al*., 2011), a temporary, extremely sensitive, and dynamic structure which participates in segregation during meiosis (Zenzes *et al*., 2001). The meiotic spindle controls chromosome movement and mediates various functions essential for fertilization and early post fertilization events (Gu *et al*., 2020).

In order to evaluate the effectiveness of Tvitri-4 for oocyte cryopreservation, we first focused on SR and meiotic spindle normality outcomes. Morphological survival can reflects the efficiency of oocyte warming and is highly associated to success in fertilization, implantation, and pregnancy in an egg donation program (Domingues *et al*., 2015). The presence of the meiotic spindle in oocytes was found to be positively associated with embryo implantation (García-Oro & Valverde, 2019). In the context of cryopreservation, spindle integrity and chromosome alignment can be significantly affected in vitrified human oocytes as a consequence of decreased ATP level. In addition, it has been reported that screening for normal meiotic spindle morphology and chromosome configuration before vitrification may increase the yield of healthy viable oocytes for various assisted reproductive technologies (Gu *et al*., 2020).

Survival rate of vitrified-warmed oocytes from F1 hybrid strains (B6.DBA and BDF-1) (Trapphoff *et al*., 2016) undergoing vitrification varies from 85.9% (Jo *et al*., 2013) to 91.8% (Kuwayama *et al*., 2005). In our study using C57BL/6J oocytes, SR as determined by oocyte morphological integrity, were 81.5% and 87.5% for ING and T4, respectively, comparable to those cited in other mouse oocyte vitrification experiments. Inversely, higher SR (92-98%) of vitrified-warmed oocytes using the combination of EG plus DMSO, and sucrose as cryoprotectants were reported in B6D2F1 mice (Gomes *et al*., 2008; Zhou *et al*., 2016). In C57BL/6J strain, Lee *et al*. (2021) reported a SR of 94.2% of vitrified-warmed oocytes. Previous studies using ING vitrification/warming media showed survival of 86% for *in vitro* matured bovine oocytes (Almodin *et al*., 2008) and 84.9% for mature human oocytes (Almodin *et al*., 2010). Clinical studies from vitrified/warmed human oocytes demonstrate SR of 67 to 100% using standard vitrification media (Almodin *et al*., 2010; Practice Committees of the American Society for Reproductive Medicine & the Society for Assisted Reproductive Technology, 2013; Morishima *et al*., 2017), variability above that demonstrated in mouse oocytes, which may be due to numerous factors other than just those related to the vitrification/warming process, such as the different ovarian stimulation protocols and the characteristics of women (age, ovarian reserve, diseases possibly related to infertility, among others) (Rienzi *et al*., 2017).

Cryopreservation may affect mitochondrial functionality, induce oxidative stress, and thereby affect spindle integrity, chromosome segregation and the oocyte quality.

Studies have been reported that the meiotic spindle microtubules depolymerize before or during the vitrification process but can repolymerize and form normal structural and functional spindles post-cryopreservation after a recovery culture (Gook & Edgar, 2007; Koutlaki *et al*., 2006; Martínez-Burgos *et al*., 2011; Varghese *et al*., 2009). In addition, a relation between spindle normality and chromosomal configuration in vitrified-warmed oocytes and the equilibration time of vitrification was observed and affects the success rates of ART (Jung & Cheon, 2014). As pointed out, to supporting oocyte cryopreservation and long-term safety is particularly important to understand whether differences exist between meiotic stages when spindle are present (i.e. metaphase I, anaphase I, telophase I, and metaphase II) and ability of microtubules depolymerize and repolymerize with fidelity (Smith *et al*., 2011).

Although no significant differences in meiotic spindle normality were observed between T4 (63.4%) and ING (52.2%) a trend towards better indexes of meiotic normality in oocytes from T4, similar to the CTR (70.5%) needs further confirmation in a larger sample size. The rates of spindle and chromosomal configuration integrity in fresh mature oocytes of CD-1 mice range from 88.9% (Navarro *et al*., 2006) to 95% (Huang *et al*., 2008). Some studies report a normal rate of 89.6% for mature oocytes of the CD-1 strain after warming (Huang *et al*., 2008). The present study considered the standard of meiotic spindle normality for vitrified oocytes in a conventional medium the value of 71.6% (Ju *et al*., 2005) to compare the results found with those in the literature. Similar meiotic spindle normality rate was reported for vitrified-warmed oocytes from B6D2 mice (Zhou *et al*., 2016). Probably the vitrification protocol contributed to some extension to the differences observed between the two media, but the formulation certainly had an important impact. In fact, we have observed that the four modifications introduced in Tvitri-4 formulation, when this vitrification medium was designed, are responsible for its good performance since the comparison between Tvitri-4 and FujiFilm Irvine Scientific vitrification media, using the same microdroplet vitrification method for both, has revealed some superior results of Tvitri-4 according to the outcome evaluated, and a better potential to prevents membrane disturbancies (Berteli *et al*., 2020a;b).

In conclusion, the T4 medium showed satisfactory results for the oocyte vitrification without any adverse effect on survival and meiotic spindle normality in comparison to a commercially available vitrification medium. Embryo development and live birth rates however should be examined in order to validate the clinical use of Tvitri-4 medium. The generation of a 100% national technology-based product can contribute to decrease the supply chain risks of imported products, and to reduce the cost of assisted reproduction procedures, increasing the accessibility of infertile couples and women desiring to cryopreserve oocytes to assisted reproduction care.
